# Modelling the population effectiveness of the national seasonal influenza vaccination programme in Scotland: The impact of targeting all individuals aged 65 years and over

**DOI:** 10.1111/irv.12583

**Published:** 2019-06-05

**Authors:** Stephen Corson, Chris Robertson, Arlene Reynolds, Jim McMenamin

**Affiliations:** ^1^ Department of Mathematics and Statistics University of Strathclyde Glasgow UK; ^2^ Health Protection Scotland Glasgow UK

**Keywords:** effectiveness, influenza, modelling, population, statistics, vaccine

## Abstract

**Background:**

For the last 17 years, the UK has employed a routine influenza vaccination programme with the aim of reducing the spread of seasonal influenza. In mid‐2000, the programme moved from a purely risk‐based approach to a risk and age group‐targeted approach with all those aged 65+ years being included. To date, there has been no assessment of the population effectiveness of this age‐targeted policy in Scotland.

**Objectives:**

Statistical modelling techniques were used to determine what impact the routine vaccination of those aged 65+ years has had on influenza‐related morbidity and mortality in Scotland.

**Methods:**

Two Poisson regression models were developed using weekly counts of all‐cause mortality, cause‐specific mortality and emergency hospitalisations for the period 1981‐2012, one using week‐in‐year and the other using temperature to capture the seasonal variability in mortality/hospitalisations. These models were used to determine the number of excess deaths/hospitalisations associated with the introduction of the local risk and age‐based vaccination programme in 2000.

**Results:**

Routinely vaccinating those aged 65+ years is associated with a reduction in excess all‐cause mortality, cardiovascular and COPD‐related mortality and COPD‐related hospitalisations. Our analysis suggests that using the week‐in‐year model, on average, 732 (95% CI 66‐1398) deaths from all causes, 248 (95% CI 10‐486) cardiovascular‐related deaths, 123 (95% CI 28‐218) COPD‐related deaths and 425 (95% CI 258‐592) COPD‐related hospitalisations have been prevented each flu season among the those aged 65+. Similar results were found using the temperature model. There was no evidence to suggest that the change in policy was associated with reductions in influenza/pneumonia‐related mortality or influenza/cardiovascular‐related hospitalisations.

**Conclusions:**

Routinely vaccinating those aged 65+ years appears to have reduced influenza‐related morbidity and mortality in Scotland. With the childhood vaccination programme well underway, these data provide an importance benchmark which can be used to accurately assess the impact of this new seasonal influenza vaccination programme.

## 1 INTRODUCTION

Every winter, the UK experiences a seasonal influenza epidemic that affects the morbidity and mortality of thousands of its citizens and puts increased pressure on NHS resources.^1^, ^2^ For healthy individuals, influenza is a self‐limiting, though debilitating, illness from which full recovery is usually attained within 2‐7 days.^2^, ^3^ There are some subsets of the population, however, who have been shown to have a higher risk of influenza associated morbidity and mortality.^4^, ^5^, ^6^, ^7^, ^8^, ^9^ Although the extent to which influenza increases morbidity and mortality varies from year to year, the virus continues to impose a considerable economic burden on society.^10^, ^11^ As a result, seasonal influenza epidemics are considered to be a significant annual public health threat.

Since the late 1960s, the UK has sought to limit the healthcare burden associated with influenza via a national vaccination programme. The programme initially targeted individuals who were at highest risk of influenza‐related morbidity and mortality and was continually expanded to incorporate a wider range of risk groups.^2^, ^3^, ^4^ In 2000, the programme was extended to include all persons aged 65+ years, moving the UK's vaccination policy from a risk based to a risk and age‐based strategy. More recently, in 2012, the Joint Committee on Vaccination and Immunisation (JCVI) recommended the inclusion of those aged 2‐17 years in the routine vaccination programme by offering intranasally administered live cold‐adapted influenza vaccine (LAIV)—Fluenz.^12^ Each of the constituent countries within the United Kingdom endorsed this JCVI recommendation which has since become government policy. The phased introduction of this new extension began in 2013 with the routine immunisation of children aged 2‐3 years with extensions to incorporate older age groups in subsequent years.^13^, ^14^ Although healthy children are less likely to experience severe influenza‐related morbidity and mortality,^15^ they are two to three times more likely to be ill with influenza^14^ and are a well‐documented transmitter of the virus.^16^, ^17^ Previous transmission modelling and cost‐effectiveness studies suggest the JCVI's recommendations will increase the overall efficiency of the influenza vaccination programme^18^ and offer a highly cost‐effective way of providing this risk group with direct protection against the impact of flu.^19^ The reduction in flu circulation resulting from the vaccination of children should offer indirect protection to older adults and those with clinical risk factors, therefore reducing the number of severe flu cases and flu‐related deaths in this subset of the population.^20^, ^21^, ^22^, ^23^

A clear understanding of how changes to the UK's vaccination programme has affected influenza‐related morbidity and mortality is key to providing a benchmark from which policymakers can assess the success of the vaccination programme. While there is evidence to suggest that the influenza vaccine is effective at preventing influenza illness and its complications,^24^, ^25^, ^26^, ^27^, ^28^, ^29^, ^30^ the population effectiveness of the UK vaccination programme is not fully understood. While previous modelling work in England suggests that the vaccination programme, which targets clinical risk groups as well as individuals aged 65+ years, is cost‐effective^24^ and associated with a reduction in the incidence of laboratory confirmed influenza illness^24^ as well as reduced levels of pneumonia and influenza‐related mortality,^31^ the same knowledge does not exist for Scotland. Understanding how many deaths/hospitalisations have been prevented among the over 65s is an important first step in this direction. Such work would (i) provide an estimate of the population effectiveness of the vaccination programme following the mid‐2000 extension and (ii) allow a comparison with similar analyses using more recent data so that healthcare professionals can assess the changes in vaccination effectiveness following the 2013/14 pilot and subsequent extension to all 2‐ to 11‐year‐olds.

The aim of this analysis was to use routine influenza surveillance data collected in Scotland to develop models that can be used to evaluate the population effectiveness of the national seasonal influenza vaccination programme. In particular, we are going to estimate the number of excess deaths/hospitalisations that may be been prevented by the mid‐2000 policy recommendation to routinely vaccinate all persons aged 65+ years.

## 2 METHODS

### 2.1 Data sources

We use yearly mid‐year estimates of population size along with data on mortality and emergency hospitalisations for this analysis. Weekly counts of all‐cause mortality, cause‐specific mortality and emergency hospitalisations in Scotland from 1981 to 2012 were provided by Health Protection Scotland. These data were extracted from the ACaDMe (Acute, Cancer, Deaths and Mental Health) datamart at Public Health Intelligence (PHI) which contains linked inpatient and daycase (SMR01), mental health (SMR04), cancer registration (SMR06) and death (GRO) records from 1981 to the present day.^32^ The cause‐specific mortality data list the number of influenza, pneumonia, cardiovascular (diseases of the circulatory system); COPD (chronic lower respiratory disease, lung diseases due to external agents and other respiratory diseases) and trauma (hernia, injury, poisoning, diseases of the appendix and other external causes)‐related deaths recorded each week for the period of interest. The emergency hospitalisation data record the number of emergency hospital admissions for each patient in Scotland and uses the same disease categories as the cause‐specific mortality data. Mid‐year estimates of population size for the period of interest were obtained from the National Records of Scotland.^33^ All of these data were broken down by gender (male or female) and age category (0‐1, 2‐4, 5‐12, 13‐17, 18‐44, 45‐64, 65‐74, 75‐84 and 85+ years).

Weekly spatial temperature data for the geographical centre of Scotland are used to account for the direct effect on temperature on the seasonal variation in mortality and hospitalisations. We use data obtained from the British Atmospheric Data Centre^34^ along with Shepard's inverse distance weighting methods^35^ to estimate weekly minimum and average air temperatures for the period of interest.

### 2.2 Modelling the number of deaths/hospitalisations for a given week in the year

We use generalised additive models (GAMs) to derive Poisson regression models to estimate the expected number of deaths/hospitalisations for a given week in the year.^36^, ^37^

We use two different GAMs in this analysis. The first GAM assumes that the seasonal variability in mortality/hospitalisations is described solely by the time of year by defining a variable that represents the location of the mid‐point of a given week as a proportion of the number of weeks in that year. We denote this model the Week‐In‐Year (WIY) model. The second GAM assumes that the seasonal variability in mortality/hospitalisations is described solely by changes in temperature. We denote this model the seasonal temperature (ST) model. Both models contain an offset term to allow for changes in the population size/number of hospitalisations over time. When analysing mortality, the offset is equal to the logarithm of the population at risk. This offset is not suitable for the analysis of hospitalisations since the number of hospitalisations per head of population (population at risk offset) changes more rapidly than the number of hospitalisations per head of hospitalised population (offset equal to the logarithm of the total number of hospital admissions). Therefore, when analysing hospitalisations, the offset is equal to the logarithm of the total number of emergency hospitalisations.

Both models are fitted to a dataset which contains all data from 1981 to mid‐2000 and summer data from mid‐2000 to 2012. Here, summer refers to be the fixed period covering weeks 10‐45 of any given year, roughly March to October, thus ensuring that there were a sufficient number of weeks for the winter season for the model. A stepwise regression method is used to derive the best fitting models. Predictor variables (age category, gender, year, week‐in‐year, flu season, minimum temperature and temperature severity) are either added to or removed from the regression equation depending on which step would provide a significant increase in the model fit (measured by the generalised cross‐validation [GCV] score). Here, week‐in‐year is a continuous variable that denotes the location of the mid‐point of a given week as a proportion of the number of weeks in that year, and minimum temperature is a continuous variable that denotes the weekly minimum air temperature for the geographical centre of Scotland. Temperature severity is a categorical variable with five levels (0‐4) as shown in Table 1 that records how mild or severe the weather was during a particular flu season. The cut‐off values of −0.6°, −1.27° and −2.5° correspond to the 1st, 2nd and 3rd quartiles of the data set that contained the negative weekly minimum air temperatures.

**Table 1:** Cut‐off values used to define the temperature severity variables. The cut‐off values of −0.6°, −1.27° and −2.5° correspond to the 1st, 2nd and 3rd quartiles of the dataset that contained the negative weekly minimum air temperatures

Severity value	Minimum air temperature (*x*)
0	*x* ≥ 0°
1	−0.6° ≤ *x* < 0°
2	−1.27° ≤ *x* < −0.6°
3	−2.5° ≤ *x* < −1.27°
4	*x* < −2.5°

We find that the best fitting WIY GAM is a function of year, week‐in‐year, age category and gender that models the number of deaths/hospitalisations in a given week *t*, for age group *a* and sex *s* (*Count*_*tas*_) using the following equation:

$$\begin{array}{cc} \text{log}(Count_{\mathrm{tas}})\;= & \;\text{offset}\;+\;\beta_{0}\;+\;f_{1}\left(year\right)\\ & \;+\;\beta_{2}gender\;+\;\beta_{3}age\;+\;f_{2}\left(week\text{‐}in\text{‐}year\right) \end{array}$$

where *f*_*i*_ denotes a cubic regression spline. Similarly, we find that the best fitting ST GAM is a function of year, minimum temperature, gender, age category and temperature severity that models the number of deaths/hospitalisations using the following equation:

$$\begin{array}{cc} \text{log}(Count_{\mathrm{tas}})\; & =\;\text{offset}\;+\;\beta_{0}\;+\;f_{1}\left(year\right)\;+\;\beta_{2}gender\\ & \;+\;\beta_{3}age\;+\;f_{2}\left(mintemp\right)\;+\;severity. \end{array}$$

Both GAMs model the general trend of decreasing mortality over time using separate cubic regression splines for each gender, with 10‐15 knot points (equivalent to one every 2‐3 years), and the seasonal variability in mortality/hospitalisations using a cyclic cubic regression spline with 20 knot points (equivalent to one every 2.5 weeks). The WIY (ST) model uses week‐in‐year (minimum temperature) to capture the seasonal variability in mortality. We follow the general purpose approach described in the documentation for the R package *mgcv* 38 to identify the correct number of knots for each spline.

The best fitting GAMs are used to predict the weekly number of deaths/hospitalisations for the period 1981‐2012. These predictions correspond to the number of deaths/hospitalisations that would be expected under the pre‐mid‐2000 (risk based) vaccination policy. For each outcome of interest, we calculate the number of excess observations (defined as observed count minus expected count) for each flu season (week 46‐week 9). To quantify the change in policy's impact on excess deaths/hospitalisations, we follow the work of Mann et al^31^ and fit a Gaussian regression model to these data. For the ST model, we use a model that contains a dummy variable that takes the value 0 for 1981‐mid‐2000 and 1 for mid‐2000‐2012 to capture the mid‐2000 change in vaccination policy. For the WIY model, we use the same dummy variable as the ST model as well as a second variable that accounts for the minimum air temperature observed in each flu season.

All the analysis is carried out using R version 3.10,^39^ and over‐dispersion is tested for using the over‐dispersion test provided by the *qcc* R package.^40^ A 5% significance level is used throughout, and 95% confidence intervals are based upon a normal distribution.

### 2.3 Sensitivity analysis

We conduct a sensitivity analysis using the ST model for those aged 65+ years to investigate how our model fits and estimates for the number deaths/hospitalisations prevented are affected when we (i) use minimum instead of average air temperature to define seasonality, and (ii) allow the flu season to vary from year to year corresponding to the national influenza surveillance data on consultation rates (reports per 100 000 population) from Health Protection Scotland. Each flu season starts when the consultation rate exceeds 50 and ends when it falls below 50.

## 3 RESULTS

### 3.1 All‐cause mortality

Weekly all‐cause mortality for those aged 65+ years in Scotland for the period 1981‐2012 ranged from 692 deaths (2010 week 29) to a maximum of 1930 deaths (1989 week 50). The maximum number of deaths recorded under the risk‐based vaccination policy (pre‐mid‐2000) was 1930 while the maximum number of deaths under the risk and age‐based vaccination policy (post‐mid‐2000) was 1192 (2007 week 2). Females aged 75‐84 (85+) years were the greatest contributors to the pre‐ (post‐) policy change maxima (n* = *457, 288, respectively).

**Figure 1:**
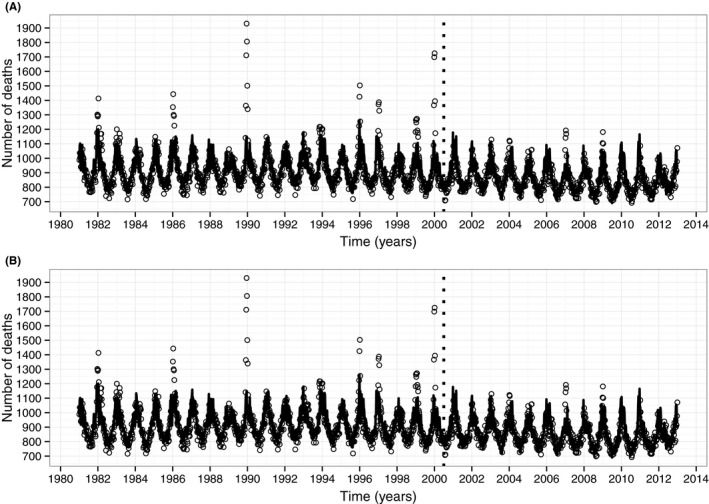
Observed weekly all‐cause mortality for those aged 65 and over (circles) with (A) WIY model predictions (solid black line), and (B) ST model predictions (solid black line). The dotted vertical line indicates the change in policy from a risk‐based vaccination strategy to a risk and age‐based vaccination strategy

**Figure 2:**
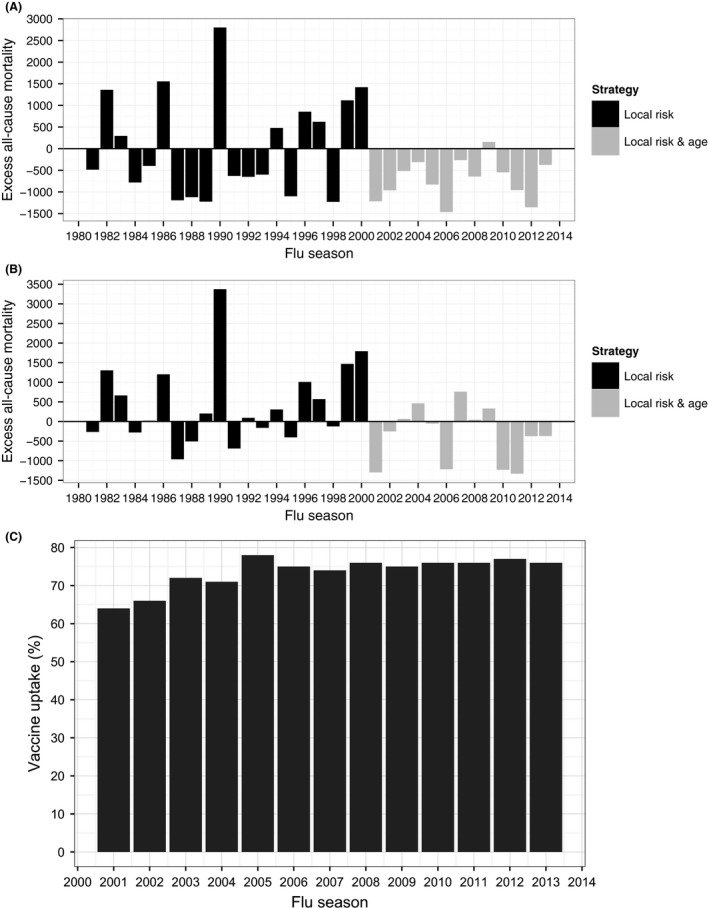
Excess all‐cause mortality (defined as observed minus predicted) for those aged 65 and over by flu season. Estimates obtained with (A) WIY model predictions, and (B) ST model predictions. Figure (C) shows the vaccine uptake data that is available from 2001

From Figure 1, we can see that although both models are able to capture the long‐term trends and the seasonal variation in the data, there are a number of instances where the models are not able to predict the number of deaths observed in moderate/severe influenza epidemics (e.g. 1989‐1990 and 1999‐2000). Excess all‐cause mortality for each flu season is shown in Figure 2 where it is evident that, prior to the policy change in 2000, there are instances where the model over‐predicts (negative excess all‐cause mortality) and under‐predicts (positive excess all‐cause mortality). This is a consequence of using both winter and summer data to fit the models prior to 2000. After the policy change, however, there are few instances of under‐prediction and many more instances of over‐prediction, suggesting that excess mortality has decreased in the period following the change in vaccination policy. It is worth noting that the model fits for the ST model were slightly better than those for the WIY model.

There is evidence to suggest that there has been a significant reduction in excess all‐cause mortality among those aged 65+ years following the mid‐2000 change in vaccination policy (WIY: *P *=* *.019, ST: *P *=* *.025) with both models predicting similar estimates of deaths prevented each flu season (WIY: 732 [95% CI 66‐1398]; ST: 775 [95% CI 131‐1418]) as shown in Table 2. Analysis of the three age groups that make up the 65+ category (65‐74, 75‐84, and 85+) suggests that significant reductions in all‐cause mortality have been observed in each of these age groups (WIY: *P *=* *.018; .034; .004; ST: *P *=* *.005; .020; .020, respectively). The greatest reductions have been among those aged 85 and over while the smallest reductions have been among those aged 65‐74 (Table 2).

**Table 2:** Estimates for the reduction in all‐cause mortality among those aged 65+ years following the mid‐2000 change in policy from a risk‐based vaccination strategy to a risk and age‐based vaccination strategy

	Reduction^a^ (WIY model)		Reduction^a^ (ST model)	
Age group	Mean	95% CI	Mean	95% CI
65+	732	66‐1398	775	131‐1418
65‐74	179	29‐330	216	75‐358
75‐84	293	21‐565	327	67‐588
85+	384	130‐637	330	69‐594

CI, confidence interval, based on a normal distribution.

a The number of deaths in the individual age categories may not sum to the total due to rounding errors.

### 3.2 Cause‐specific mortality

Among those aged 65+ years, mortality attributable to influenza, pneumonia, cardiovascular disease, COPD and trauma were highly variable. Influenza‐related mortality ranged from 0 to 139 deaths (1989 week 50) while pneumonia, cardiovascular, COPD and trauma‐related mortality ranged from 17 (2012 week 29) to 297 (1989 week 50), 183 (1989 week 34) to 771 (1989 week 51), 16 (1983 week 34) to 180 (2000 week 1) and 0‐65 (2000 week 1) deaths, respectively. Recorded instances of influenza‐related mortality were relatively rare: only 321 (30.7%) of the 1044 time points pre‐policy change and 55 (8.8%) of the 626 time points post‐policy change contained nonzero entries.

The model predictions for the 65+ grouping are presented in Table 3. As expected, there is no evidence of a reduction in trauma‐related mortality following the change in vaccination policy. Furthermore, there is no evidence of a reduction in influenza and pneumonia‐related mortality. Our analysis does, however, find evidence to suggest that there has been a significant reduction in cardiovascular (WIY: 248 [95% CI 10‐486]; ST: 310 [95% CI 105‐515]) and COPD (WIY: 167 [95% CI 75‐259]; ST: 123 [95% CI 28‐218])‐related mortality (Table 3).

**Table 3:** Estimates for the reduction in influenza, pneumonia, cardiovascular, COPD and trauma‐related deaths following the mid‐2000 change in policy from a risk‐based vaccination strategy to a risk and age‐based vaccination strategy. Confidence intervals that contain zero indicate mortality categories that were not affected by the change in vaccination policy

	Reduction^a^^,^ b (WIY model)		Reduction^a^^,^ b (ST Model)	
Age group	Mean	95% CI	Mean	95% CI
Influenza‐related mortality				
65+	10	−50‐70	15	−37‐66
65‐74	0	−9‐9	1	−8‐10
75‐84	3	−21‐27	6	−15‐27
85+	6	−20‐32	8	−16‐31
Pneumonia‐related mortality				
65+	62	−104‐229	85	−60‐236
65‐74	8	−17‐33	13	−9‐35
75‐84	12	−52‐77	26	−32‐84
85+	51	−30‐132	54	−20‐127
Cardiovascular‐related mortality				
65+	248	10‐486	310	105‐515
65‐74	58	18‐91	82	25‐139
75‐84	108	5‐212	138	53‐223
85+	110	22‐197	116	32‐200
COPD‐related mortality				
65+	167	75‐259	123	28‐218
65‐74	55	18‐91	45	10‐81
75‐84	64	21‐107	49	2‐95
85+	40	21‐59	25	3‐48
Trauma‐related mortality				
65+	−6	−28‐15	−4	−28‐19
65‐74	−6	−14‐2	−5	−13‐3
75‐84	−8	−18‐2	−7	−17‐3
85+	−11	−24‐1	10	−5‐25

CI, confidence interval, based on a normal distribution.

a The number of influenza, pneumonia, cardiovascular, COPD, and trauma‐related deaths prevented each flu season.

b The number of deaths in the individual age categories may not sum to the total due to rounding errors.

### 3.3 Cause‐specific emergency hospitalisations

Influenza, pneumonia, cardiovascular disease, COPD and trauma‐related hospitalisations among those aged 65+ years were highly variable. Emergency hospitalisations attributable to influenza ranged from 0 to 88 (2000 week 1) while those attributable to pneumonia, cardiovascular, COPD and trauma ranged from 24 (1982 week 32) to 407 (1999 week 52), 353 (1982 week 29) to 934 (1995 week 52), 45 (1983 week 43) to 666 (1999 week 52) and 188 (1981 week 39) to 740 (2008 week 49), respectively.

Estimates for the number of hospitalisations prevented among the 65+ grouping are presented in Table 4. Our analysis shows that there is no evidence to suggest that the change in policy has had an effect on trauma, influenza and cardiovascular‐related hospitalisations. There is, however, evidence to suggest that there has been a significant reduction in COPD‐related hospitalisations among those aged 65+ years (WIY: 425 [95% CI 258‐592]; ST: 236 [95% CI 40‐431]). The greatest reductions in COPD hospitalisations have been among those aged 65‐74 while the smallest reductions have been among those aged 85+ years. Furthermore, the two models produce slightly contradictory results when we analyse pneumonia‐related hospitalisations: the WIY model finds evidence of a significant reduction in these hospitalisations (245, 95% CI 104‐385) while the ST model does not (147, 95% CI −4‐298).

**Table 4:** Estimates for the reduction in influenza, pneumonia, cardiovascular, COPD and trauma emergency hospitalisations following the mid‐2000 change in policy from a risk‐based vaccination strategy to a risk and age‐based vaccination strategy. Confidence intervals that contain zero indicate hospitalisation categories that were not affected by the change in vaccination policy

	Reduction^a^^,^ b (WIY model)		Reduction^a^^,^ b (ST Model)	
Age group	Mean	95% CI	Mean	95% CI
Influenza‐related hospitalisations				
65+	21	−22‐65	24	−14‐62
65‐74	5	−10‐20	7	−8‐21
75‐84	10	−10‐29	10	−7‐26
85+	6	−5‐16	6	−2‐14
Pneumonia‐related hospitalisations				
65+	245	104‐385	147	−4‐298
65‐74	64	19‐109	44	−2‐91
75‐84	136	70‐202	75	5‐145
85+	41	4‐78	14	−27‐55
Cardiovascular‐related hospitalisations				
65+	42	−118‐202	52	−107‐211
65‐74	10	−64‐84	15	−57‐87
75‐84	25	−48‐98	14	−57‐86
85+	13	−29‐56	43	0‐85
COPD‐related hospitalisations				
65+	425	258‐592	236	40‐431
65‐74	201	111‐290	115	9‐221
75‐84	167	99‐234	94	21‐167
85+	53	28‐78	25	0‐85
Trauma‐related hospitalisations				
65+	83	−51‐216	−41	−153‐72
65‐74	26	−38‐90	−34	−88‐21
75‐84	6	−54‐66	−41	−94‐13
85+	−11	−3‐69	27	−9‐63

CI, confidence interval, based on a normal distribution.

a The number of influenza, pneumonia, cardiovascular, COPD and trauma‐related hospitalisations prevented each flu season.

b The number of hospitalisations in the individual age categories may not sum to the total due to rounding errors.

### 3.4 Sensitivity analysis

Our sensitivity analyses results are presented in Table 5. For all cases, changing the temperature measure to minimum temperature results in a decrease in model fit. Improved model fits were observed for influenza and pneumonia‐related mortalities as well as influenza and cardiovascular‐related hospitalisations when we allowed the flu season to vary year on year. These improved models result in increases in the number of (i) influenza‐related deaths prevented (from 15 to 24), (ii) pneumonia‐related deaths prevented (from 85 to 96), and (iii) cardiovascular‐related hospitalisations prevented (from 52 to 56). It is worth noting that the changes in model fits did not alter the statistical significance of any reductions in mortality/hospitalisations. These sensitivity analyses did not produce any substantial changes to the impact of the vaccination campaign.

**Table 5:** Sensitivity analysis results showing the GCV scores and the mean number of deaths and hospitalisations prevented among those aged 65 and over. For each outcome of interest, smaller GCV scores mean better model fits

	Baseline		Min temp^a^		Flu seasons^b^	
Cause	GCV score	Reduction^c^	GCV score	Reduction^c^	GCV score	Reduction^c^
Deaths						
All‐cause	2.397	775	2.666	811	2.589	782
Influenza	0.660	15	0.661	17	0.658	20
Pneumonia	1.596	85	1.622	99	1.574	96
Cardiovascular	2.196	310	2.235	351	2.198	324
COPD	1.456	123	1.477	111	1.462	114
Hospitalisations						
Influenza	0.698	24	0.700	19	0.686	13
Pneumonia	1.415	147	1.426	105	1.421	74
Cardiovascular	1.054	52	1.057	24	1.052	56
COPD	1.778	236	1.796	175	1.784	125

a Model uses the minimum temperature for the geographical centre of Scotland to define seasonal variability. Predictions are also adjusted for severity of temperature.

b Model uses consultation rates (reports per 100 000 population) to define the start and end of each flu season.

c The mean number of events prevented each flu season since mid‐2000.

## 4 CONCLUSIONS AND DISCUSSION

For the last 17 years, the UK has employed a routine influenza vaccination programme with the aim of reducing the spread of seasonal influenza.^41^ The programme has been extended to allow for the introduction of new risk groups and, in mid‐2000, moved from a purely risk‐based approach to a risk and age group‐targeted approach with all those aged 65+ years being included.^42^ The 2012 JCVI recommendations to include 2‐ to 17‐year‐olds^12^ is a major undertaking that will require a substantial increase in NHS resources. To date, there has been no assessment of the population effectiveness of the age‐targeted policy introduced in Scotland in 2000. Understanding how that policy extension has affected influenza‐related morbidity and mortality is key to understanding the effectiveness of the current extension.

In this article, we have used routine influenza surveillance data collected in Scotland for the period 1981‐2012 to develop Poisson regression models to evaluate the population effectiveness of Scotland's national seasonal influenza vaccination programme.

Our results suggest that mortality/hospitalisations among those aged 65+ years were reduced following the mid‐2000 change in policy. In particular, we found evidence of reductions in all‐cause mortality (WIY: 732; ST: 755 deaths prevented), COPD (WIY: 167; ST: 310 deaths prevented), and cardiovascular (WIY: 248; ST: 123 deaths prevented)‐related mortality, and COPD‐related hospitalisations (WIY: 425, ST: 236 hospitalisations prevented).

It is believed that this study is the only one to analyse the population impact of the influenza vaccine on several causes of mortality/hospitalisations. Since 2008, there have been a number of groups looking at the annual effectiveness of the seasonal influenza vaccine,^43^, ^44^, ^45^, ^46^ using mainly a test negative case‐control study design, and these are now used to inform the WHO strain selection meetings each year. That said, previous studies that have examined the impact on mortality of routinely vaccinating those aged 65+ years have produced contradictory results. Studies in the USA and Italy found no evidence of a reduction in all‐cause mortality among the over 65s,^47^, ^48^ while a study in Holland did find evidence of a reduction.^49^ The failure to find evidence in the USA and Italian studies may be due to a limited period with sufficiently high vaccine coverage.^31^ There are few studies in the UK that have focused on the vaccination programme among those aged 65+ years. In contrast to our results for Scotland, Mann et al^31^ found weak evidence of a reduction in influenza and pneumonia‐related mortality in England and Wales following the mid‐2000 policy change. However, their approach uses a longer definition of the flu year (week 26 to week 25), therefore incorporating deaths that occur outside the flu season. Furthermore, any estimates of negative excess deaths were recoded to 0. As a result, Mann et al could potentially overestimate the impact of the change in vaccination policy.

Data on consultation rates collected in Scotland show that the start, end and duration of the flu season can vary from year to year. This suggests that our definition of a flu season, the fixed period from week 46 to week 9, may limit our ability to capture flu seasons that start early or finish late. Sensitivity analyses showed that allowing the flu season to vary from year to year did alter our estimates for the number of deaths/hospitalisations prevented each flu season. However, the changes in our estimates were, with the exception of pneumonia and COPD hospitalisations, relatively small. The changes in our estimates for pneumonia and COPD may be attributable to the following: While pneumonia and COPD admissions are at increased levels in the winter when flu is circulating, there are other infectious agents that can cause admissions for pneumonia and COPD in the periods out with the winter. Furthermore, the model fits obtained from the varying flu season model were, for the most part, poorer than those obtained with the baseline model.

We used two different methods to account for the seasonal variation in the data. This allowed us to validate our results and test the assumption that seasonal variation in mortality/hospitalisations is associated with temperature. For all‐cause and cause‐specific mortality these two methods produced similar estimates of the vaccination campaign's impact. For cause‐specific hospitalisations, the two methods produced similar estimates of the vaccination campaign's impact for all but one of the hospitalisation categories: contradictory results for pneumonia‐related hospitalisations. Here, the WIY model found evidence of a significant reduction while the ST model did not. This apparent contradiction may be attributable to the poorer model fits that were obtained using the ST model (data not shown). The poorer fits suggest that the variability in mortality/hospitalisations cannot be explained by temperature alone. This suggests that the WIY model, which uses a generic term to capture the seasonal variation, may be the most appropriate method to use.

We have assumed throughout that any changes in the excess deaths/hospitalisations post‐2000 compared to pre‐2000 are associated with the UK's influenza vaccination programme. It is possible that the changes may be associated with features like severity of flu, the use of antibiotics or disease modification drugs such as statins. We use a dummy binary variable to introduce the routine vaccination of all those aged 65+ in mid‐2000. We assume no vaccine effect/availability prior to mid‐2000. Before mid‐2000 the UK employed a risk‐based vaccination strategy and it is likely that some of those aged 65+ would have been vaccinated as part of that programme. Therefore, there may be some vaccine effect pre‐mid‐2000 that our model does not account for which may lead to an overestimation of the effectiveness of the age‐based vaccination policy. Unfortunately, yearly vaccine uptake data for Scotland is only available from 2001, after the introduction of the age‐based vaccination strategy, and we are therefore unable to adjust for vaccine uptake or use vaccine uptake as a predictor variable. There has been an increase in vaccine uptake among those aged 65+ years over the period 2001‐2012, but there is no evidence from Figure 2 of a trend to have increased reductions in the excess post‐2001 and this suggests that vaccine uptake is not an explanation. Similarly, antibiotic use has increased steadily over the post‐2001 period and the lack of a trend suggests this is not a contributing factor. Furthermore, the trends in our data may be associated with behavioural changes like reduced rates of smoking (smoking may predispose the individual to increased complication from influenza or other respiratory infections more evident in the winter). The inability to account for these other features in our model may lead to an overestimation of the amount of morbidity and mortality that has been prevented by the UK's influenza vaccination programme.

Previous analyses of excess winter deaths have use Serfling‐type models based upon sine and cosine terms to model the cyclical pattern to mortality within a year.^31^, ^50^, ^51^ With such models, where the data used to fit the model come from the summer months only, the excess deaths in winter are the difference between the observed and the deaths predicted assuming that the spring summer autumn seasonal trend carries on into winter. Such models are not appropriate for our work as (i) there is always likely to be an excess each season and (ii) they do not properly fit the winter peak. To achieve the latter, it is more appropriate to use either more harmonic terms in a Serfling‐type model or to use GAMS which have greater flexibility to fit to the winter peak. We use GAMS because we assess the impact of the vaccination campaign by fitting the model to all the pre‐vaccination data, both summer and winter, so that we have a model which, when projected into the future gives predictions of the number of deaths in winter, assuming that there had not been an intervention—the age‐related vaccination campaign. We also fit to the summer data in the intervention period so that we can fit to general trends of decreasing mortality in the intervention period. Failure to do so would mean extrapolating the general decreasing trends from 1981 to 1999 10‐13 years into the future. This is not optimal as the trends are unlikely to continue up to 10‐13 years into the future.

In conclusion, our work suggests that influenza‐related morbidity and mortality among those aged 65+ years has reduced following the decision to extend the UK's vaccination policy to incorporate all persons aged 65 and over. Given the recent recommendations to extend the programme to all those aged 2‐17, these results provide healthcare professionals and policymakers with an important benchmark from which to assess the success of the most recent recommendation. Furthermore, the methods used in this analysis provide a framework that can be used to analyse the population effectiveness of the new influenza vaccination programme once the 2‐ to 17‐year‐old extension has been implemented.
